# Current Status and Prevention Strategy for Coal-arsenic Poisoning in Guizhou, China

**Published:** 2006-09

**Authors:** Dasheng Li, Dong An, Yunsu Zhou, Jie Liu, Michael P. Waalkes

**Affiliations:** ^1^ Center for Disease Control of Guizhou, Guiyang, China; ^2^ Center for Disease Control of Southwest Guizhon; ^3^ Inorganic Carcinogenesis Section, Laboratory of Comparative Carcinogenesis, National Cancer Institute at National Institute of Environmental Health Sciences, Research Triangle Park, NC, USA

**Keywords:** Arsenic, Arsenicosis, Coal-arsenic exposure, Health effects, Neoplasms, Health education, Nutrition, Drug therapy, Review literature, China

## Abstract

Arsenic exposure from burning coal with high arsenic contents occurs in southwest Guizhou, China. Coal in this region contains extremely high concentrations of inorganic arsenic. Arsenic exposure from coal-burning is much higher than exposure from arsenic-contaminated water in other areas of China. The current status and prevention strategies for arsenic poisoning from burning high-arsenic coal in southwest Guizhou, China, is reported here. Over 3,000 arsenic-intoxicated patients were diagnosed based on skin lesions and urinary arsenic excretion. Non-cancerous toxicities and malignancies were much more common and severe in these patients than in other arsenic-affected populations around the world. The high incidence of cancer and arsenic-related mortality in this cohort is alarming. Chelation therapy was performed but the long-term therapeutic effects are not satisfactory. The best prevention strategy is to eliminate arsenic exposure. Funds from the Chinese Government are currently available to solve this arsenic exposure problem. Strategies include the installation of vented stoves, the use of marsh gas to replace coal, health education, the improvement of nutritional status, and the use of various therapies to treat arsenic-induced skin and liver diseases.

## INTRODUCTION

Inorganic arsenic is one of the most significant hazards to the world's population, particularly in the developing countries of Asia (1–4). Environmental exposure to arsenic mainly occurs through drinking-water contaminated with inorganic arsenic (1–3). Exposure also occurs through burning arsenic-containing coal (4). We report here the current status and prevention strategies for arsenic poisoning from burning high-arsenic coal in southwest Guizhou, China.

## DISTRIBUTION OF ARSENIC-CONTAINING COAL

The southwest Guizhou province is a mountainous region. A geologic process called epigenetic mineralization has caused extra-ordinarily high concentrations of arsenic (>100 ppm) in local coal in the region. High-arsenic coal co-exists with Carlin-type gold deposit, antimony, thallium, and mercury (5) geologically distributed in this mountainous region, and its use directly affects over 40,000 residents (6).

**Fig. 1.**
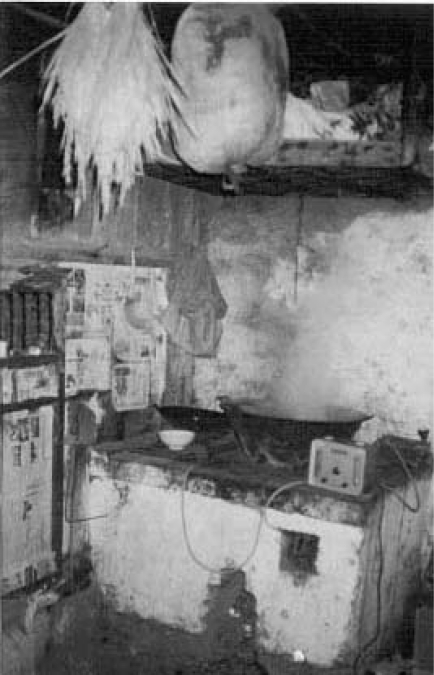
Arsenic exposure from burning arsenic-containing coal in an open stove

Coal became the main source of energy for domestic cooking and heating in the 1960s, when wood became scarce with the depletion of local natural forests. Residents frequently bring food indoors and place it above their unvented coal-burning stoves to dry (Fig. 1). Arsenic is released into the air during coal-burning, and the indoor aid concentrations of arsenic can be as high as 93–261 μg/m^3^, 31–87 times higher than the Air Quality Permission Standard of China (3 μg/m^3^). Arsenic in the air-coats and permeates the food being dried, and the arsenic content in smoke-dried food is 4.13–693 mg/kg, 6–990 folds higher than the food standard for arsenic (7). The various sources of arsenic in this endemic arsenic-poisoning area are, therefore, food (50–80%), air (10–20%), water (1–5%), and direct skin contact (<1%) (4–8, 10–11). The estimated arsenic exposure from inhalation and ingestion in this region could be up to 10 times higher than arsenic exposure through drinking-water in other parts of China (6–8).

Other elements contained in coal, such as antimony and fluorine (5–9), are also released into the air and concentrated in dried foods, which likely exposes the population to a complex mixture of potentially toxic elements. In the Guizhou region, arsenic intoxication is often accompanied with fluorosis (4–8), and thallium exposure is another health concern (9). All of these could complicate the overall toxic response. Although arsenic is clearly the main inorganic toxicant in this exposure setting, other elements are also common and likely add to the poisoning (4–8).

## EPIDEMIOLOGY OF ARSENIC INTOXICATION

Arsenic poisoning has now been reported from nine prefectures, involving 32 villages and 9,000 families in southwest Guizhou. Of 40,000 directly-exposed people, 3,000 were diagnosed with arsenic intoxication using the criteria of arsenic-exposure history, urinary arsenic excretion, and skin lesions (7–8, 10). Arsenic intoxication was observed among all age-groups; the youngest arsenic patient was aged four years, and the oldest one 78 years, with the majority of the affected people aged 20–50 years. More males than females were diagnosed as arsenic patients, and a higher intake of arsenic-contaminated food could be a reason. No occupational differences were observed in arsenic intoxication. A unique feature of arsenic intoxication in this region is individual variation. Under the same exposure conditions, there was no reported instance in which all the members of the same family showed signs of arsenic intoxication. The incidence and severity of arsenic poisoning vary greatly among the same family and among the people under the same exposure conditions, suggesting the exis-tence of susceptibility factors for arsenic intoxication in this population.

## CLINICAL MANIFESTATIONS OF ARSENIC POISONING

Skin lesions are a major clinical sign of arsenic intoxication. Approximately, 17% of the residents in the region have obvious dermal lesions. Hyperkeratosis (32% of cases) of the palms of the hands and soles of the feet and hypopigmentation (47%) in the trunk and pigmentation in the body (27%) are most common. Skin cracks and ulcers (6%) are regarded as pre-neoplastic lesions. Bowen's disease and skin cancers can be as high as 4% (8, 10). These skin lesions, once formed, show little re-versibility, even when the general health conditions of the patient improve. Thus, skin lesions can be used as diagnostic criteria but may not be a good parameter for therapeutic effects (8).

Other symptoms include blurred version (77%), numbness (69%), abdominal pain (42%), disorders of the cardiovascular system (50%), and indigestion and gastrointestinal tract symptoms (31%). Hepatomegaly (37%) is also commoner with exposure via burning arsenic-containing coal compared to arsenic exposure from contaminated drinking-water in other areas of China (4, 7). Liver cirrhosis, ascites, and probably hepatocellular carcinoma are the leading causes of death in this population exposed to arsenic through burning coal (4, 7, 11, 12).

**Fig. 2.**
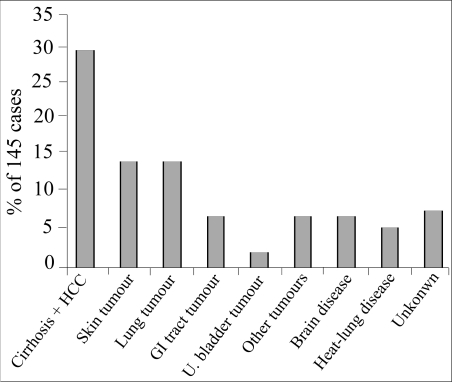
Arsenic exposure-related mortality in southwest Guizhou, China

Recent surveys (11–12) revealed that malignancies were the most serious outcome of arsenic intoxication, accounting for 50% of deaths in these arsenic arsenicosis patients (12) (Fig. 2). In another survey (11), of 123 patients (male 91, female 32) who died due to various types of cancer, lung cancer (39%), hepatocellular carcinoma (31%), and skin cancer (25%) were the most common malignancies, while stomach cancer (2%), urinary bladder cancer (2%), and colon cancer (1%) were also reported. More males than females suffered from arsenic intoxication (10–12), and the rate of mortality among arsenicosis patients was five times higher than that among the general population (77.55/10,000 vs 13.35/10,000). The death rate increased from 32/10,000 in 1991 to 113/10,000 in 2000, suggesting the urgent need to treat these arsenicosis patients and to lessen their exposure.

HCC=Hepatocellular carcinoma;
GI=Gastrointestinal;
U=Urinary

## EFFORTS TO IMPROVE THE ENVIRONMENT AND RELIEVE ARSENIC POISONING

Since 1990, chelating therapy with meso-2,3,-dimercaptosuccinic acid (DMSA), penicillamine, and/or 2,3-dimercapto-1-propanesulfonic acid (DMPS) has been used for eliminating arsenic from the body. Although the short-term effect is positive, the long-term improvement has been unsatisfactory. Chelation has little effect on severely arsenic-intoxicated patients (7–8).

Elimination of arsenic exposure is, thus, the key to the prevention of these adverse health effects. The Chinese Government has recently provided significant funding to solve these coal-burning-related health concerns, including arseniasis and fluorosis, and for installation of vented stoves and for a trial of marsh gas to replace coal. In this regard, large-scale health education is very important (7) to stop the use of arsenic-containing coal.

Since the Guizhou region is an under-developed rural area in southwest China, poor nutrition is a major concern. As in other developing countries, poor nutritional status can increase the susceptibility of an individual to chronic arsenic toxicity, and chronic arsenic intoxication could contribute to poor nutritional status (13). Thus, funds for the control of endemic arsenicosis and fluor-sis are also aimed at improving the general living standards in the region, and the solution to poverty is essential for the prevention and treatment of arsenicosis, especially of children. Protection of child health is of utmost significance in the future.

## CONCLUSION

The rate of mortality among arsenicosis patients in the Guizhou region has increased during the last 10 years, which suggests the need for urgent treatment of arsenicosis patients to prevent arsenic exposure-related deaths and carcinogenesis. Chelating therapy has not been satisfactory and, thus, the therapies directed towards various symptoms are encouraged and performed in the region. Such symptom-relieving therapies include skin lotion derived from herbs of medicinal value to soften keratosis and skin cracks and a Chinese medicine preparation—*Han-Dan-Gan-Le*—for liver disorders (14), which has been proved to be a promising method for the treatment of arsenic-induced liver fibrosis and hepatomegaly. Various therapies to treat arsenic-induced skin and liver diseases are currently under clinical trial.

In summary, coal-burning arsenic intoxication is a serious health concern. Government funds have been provided, and strategies have been put in place to reduce arsenic exposure, to improve general living conditions, and to treat various arsenic-related diseases in the heavily-exposed population of Guizhou province.

## ACKNOWLEDGEMENTS

The authors thank Dr. Jean-François Coppin, Dr. Jun Shen, and Dr. Larry Keefer for their critical review of the manuscript. Research was funded in part by the Guizhou Scientific Foundation 2001–106 and 2001–3068, China and Intramural Research Program of Center for Cancer Research, National Cancer Institute, USA.

## References

[1] National Research Council (1999). Arsenic in the drinking water.

[2] Rahman M (2002). Arsenic and contamination of drinking-water in Bangladesh: a public-health perspective (editorial). J Health Popul Nutr.

[3] Sun G (2004). Arsenic contamination and arsenicosis in China. Toxicol Appl Pharmacol.

[4] Liu J, Zheng B, Aposhian HV, Zhou Y, Chen ML, Zhang A (2002). Chronic arsenic poisoning from burning high-arsenic-containing coal in Guizhou, China. Environ Health Perspect.

[5] Finkelman RB, Belkin HE, Zheng B (1999). Health impacts of domestic coal use in China. Proc Natl Acad Sci USA.

[6] An D, Ho GY, Hu XQ (1994). Chronic arsenic-fluorine intoxication from burning coals with high arsenic and fluorine content. Chin J Prev Med.

[7] An D, Li D (2005). Epidemiological status and countermeasures of endemic arsenicosis of Guizhou province. Chin J Endemiol.

[8] Zhou YS, Zhou DX, Zheng BS, Yang DQ, Luo ML, Zhang HT (1998). Epidemiological investigation on coal-burning type of arsenic poisoning in different environments within 20 years. Chin J Endemiol.

[9] Xiao T, Guha J, Boyle D, Liu CQ, Chen J (2004). Environmental concerns related to high thallium levels in soils and thallium uptake by plants in southwest Guizhou, China. Sci Total Environ.

[10] Li D, An D, Li P, Wang SQ, Zhou D, Zhou YS (2002). Diagnostic criteria for arsenism from coal-burning in Guizhou. Chin Clin Med.

[11] Li D, An D, Zeng Z, Zhu A-H, Zhang R-Z (2004). Mortality of malignant tumor patient with endemic arsenism caused by coal-burning pollution in Guizhou. Chin J Endemiol.

[12] Zhou YS, Du H, Cheng M-L, Liu J, Zhang XJ, Xu L (2002). The investigation of death from diseases caused by coal-burning type of arsenic poisoning. Chin J Endemiol.

[13] Milton AH, Hasan Z, Shahidullah SM, Sharmin S, Jakariya MD, Rahman M (2004). Association between nutritional status and arsenicosis due to chronic arsenic exposure in Bangladesh. Int J Environ Health Res.

[14] Lu T, Cheng M-L, Wu J, Deng K-S, Zhou Y-S, Waalkes MP (2000). The therapeutic effects of Chinese medicine, *Han-Dan-Gan-Le*, on arsenic-induced liver disorders in Guizhou, China (abstract). Hepatology.

